# A case-control study on the dietary taurine intake, nutrient status and life stress of functional constipation patients in Korean male college students

**DOI:** 10.1186/1423-0127-17-S1-S41

**Published:** 2010-08-24

**Authors:** Jeong-Soon You, Ji-Yeon Park, Kyung-Ja Chang

**Affiliations:** 1 Dept. of Food and Nutrition, Inha University, Incheon 402-753, Korea

## Abstract

**Background:**

Constipation is a common gastrointestinal symptom in Korea as well as in Western countries. This study was performed to investigate the dietary taurine intake, nutrient status, and life stress of functional constipation (FC) patients in Korean male college students.

**Methods:**

Research data were collected in 2008 and a total of 104 male students (52 with FC patients and 52 healthy controls without FC) were included. FC patients were defined by the codes for the RomeⅡ Modular Questionnaire and healthy controls without FC were matched for age, height, weight and BMI. A self-administered life stress score and 3-day recall method were used to assess life stress level and dietary intake, respectively.

**Results:**

The averages of age, height, weight, body fat percentage and body mass index (BMI) of male students were 23.4 years, 174.1 cm, 71.9 kg, 19.0 % and 23.7 kg/m^2^, respectively. Average intake of dietary taurine was 126.8 mg/day in FC patients and 105.1 mg/day in control group. The average intake of total calorie (p<0.05), plant protein (p<0.01), plant fat (p<0.001), carbohydrate (p<0.05), plant calcium (p<0.05) of FC patients were significantly higher compared to control group. The average total life stress score (p<0.01), economy problem score (p<0.05), future problem score (p<0.05) and value problem score (p<0.05) of FC patients were significantly higher compared to control group.

**Conclusions:**

These results may suggest that FC patients show a higher life stress score and intake of some nutrient such as total calorie, plant protein, plant fat, carbohydrate and plant calcium in Korean male college students. Therefore, a further large-scale study is needed about correlation between life stress and nutrients intake including dietary taurine.

## Background

Functional constipation is a common gastrointestinal functional disorder and occurs in 12~27%[1-3] of people depending on demographic factors and definition. Functional constipation(FC) is a “functional bowel disorder that presents as persistently difficult, infrequent, or seemingly incomplete defecation, which do not meet irritable bowel syndrome criteria.” [4] The definite cause of functional constipation is unclear, but it is influenced by socioeconomic level, gender, lifestyle, physical activity and dietary pattern [5,6]. In addition, psychological abnormalities are also a contributory factor. It has been reported that constipation is associated with stress in children, adolescents [7] and female adults [8].

Dietary intake of taurine may play a significant role in physical and psychological well-being. It has been shown that dietary taurine intake reduced life stress level [9]. There are many previous reports about the relationship between constipation and nutrient intake [10-12]. Insufficient fiber intake and water intake, low calorie intake, or food sensitivities may contribute to the pathogenesis of constipation. However, there exists only a limited amount of the studies on dietary taurine intake and the relationship between nutrient intake and life stress in FC patients. Therefore, we investigated the dietary taurine intake and the nutrient status of FC patients and the correlation between nutrients intake including dietary taurine and life stress scores through a case-control study in Korean male college students.

## Methods

### Subjects

The subjects were male college students residing in the Incheon area and attending a nutrition-related non-major class via the internet. A case-control study was carried out using a self-administered questionnaire. The FC patients group consisted of 52 male college students that were diagnosed with functional constipation and self-reportedly claimed constipation. The control group was made up of 52 male college students who were not diagnosed with functional constipation and did not self-reportedly claim constipation. The healthy controls were matched with FC patients for age, height, weight and BMI. FC was diagnosed by the Rome Ⅱcriteria [13]. Rome Ⅱ criteria included 12 weeks or more of symptoms in the preceding 12 months, including hard or lumpy stools, straining, a sense of incomplete evacuation, the need to use manual maneuvers to pass stool, or a sense of anorectal obstruction with ≥25% of bowel movements, and/or <3 bowel movements/week, with no evidence of organic disease. At least two symptoms should be present to make the diagnosis of functional constipation.

### General characteristics and anthropometric measurement

Questions for general characteristics included 3 items such as age, type of residence and pocket money. The height of subjects was measured with a stadiometer. Body weight, body fat percentage and body mass index (BMI) were determined with an InBody 3.0 Body Composition Analyzer (InBody 3.0, Biospace, Korea).

### Dietary taurine and nutrients intake assessment

Dietary intake was assessed by a 3-day recall method (2 weekdays and 1 weekend day). Dietary taurine and nutrients intakes were estimated using the computer-aided nutrition program (CAN-pro 3.0, The Korean Nutrition Society, Korea) inputted with a taurine content database for 17 food groups and 321 commonly used food items [14,15].

### Life stress score

A self-administered life stress questionnaire was used to assess life stress level, which contained fifty questions about the frequency of stress experienced and the importance of the stress for the past year. The scores were based on a 4-point rating scale of 0 to 3 [16]. Life stress scores for college students consisted of eight life stress categories: (interpersonal relationship; faculty, lover, friend, family), (task-related stress; school grade, future, economy, value). The total life stress score was calculated by multiplying the frequency of stress experienced by the importance of stress. The higher stress scores meant the more frequency of stress experienced and the higher importance of stress.

### Statistical analysis

Data were analyzed with the SPSS 17.0 program. The frequency and percentage, and the mean and standard error for each survey question were calculated. We used the chi-square test and Student’s t-test to compare differences between FC patients and control group. The correlation between life stress scores and nutrients intake was analyzed using Pearson's correlation coefficient.

## Results and discussion

### General characteristics

General characteristic data of the subjects are shown in Table 1. The averages of age, height, weight, percent body fat and BMI of the subjects were 23.4 years, 174.1 cm, 71.9 kg, 19.0% and 23.7 kg/m^2^, respectively. Thirty-three FC patients (66.0%) and 28 controls (54.9%) lived with their families, and 36 FC patients (70.6%) and 28 controls (54.9%) spent 200,000 ~ 400,000 Korean won (approximately $175.00 ~ $350.00 US) per month for pocket money.

**Table 1:** General characteristics and anthropometric measurements of the subjects

Variables	FC patients (n=52)	Control (n=52)
Age (years)	23.3 ± 0.3^NS^	23.4 ± 0.3
Height (cm)	173.8 ± 0.7	174.6 ± 0.8
Weight (kg)	71.7 ± 1.4	72.1 ± 1.4
Body fat percentage (%)	18.9 ± 0.7	19.2 ± 0.7
BMI (kg/m^2^)	23.7 ± 0.4	23.6 ± 0.4
Type of residence		
Living with family	28(54.9)	33(66.0)
Preparation of own meals	15(29.4)	16(32.0)
Dormitory	5(9.8)	0(0)
Boarding or relatives home	3(5.9)	1(2.0)
Pocket money (1,000 Korean won/month)		
< 200	13(25.5)	6(11.8)
200 - 400	28(54.9)	36(70.6)
≥ 400	10(19.6)	9(17.6)

Values are mean ± SE and number (%); NS: not significant; BMI: body mass index

### Intakes of dietary taurine and nutrients

Average intake of dietary taurine is shown in Table 2. Average intake of dietary taurine in FC patients and control group were 126.8 and 105.1 mg/day, respectively. There was no significant difference in dietary taurine intake between FC patients and control group. The dietary taurine intake of the Korean male college student in 2006 was 124.1 mg/day in same area where this study was conducted [9] and that of Japanese male adults was 222.5 mg/day in Hokuriky District where fish and shellfish intake was the third highest among the 9 districts of Japan [17].

**Table 2:** Dietary taurine intake of the subjects

Variables	FC patients (n=52)	Control (n=52)
Taurine (mg/day)	126.8 ± 12.6^NS^	105.1 ± 7.6

Values are mean ± SE, NS: not significant

Average energy intake in FC patients and control group were 1876.2 and 1695.2 kcal/day, respectively (Table 3). There were significant differences between FC patients and control group in total calorie (p<0.05), plant protein (p<0.01), plant fat (p<0.001), carbohydrate (p<0.05) and plant calcium (p<0.05). Since eating activates colon motor function by gastrocolic reflex [18], it has been reported that constipated persons had a lower calorie and nutrient intake than non-constipated persons [19], and that dietary fiber increased fecal bulk and improved bowel habits [20]. However, in a large cross-sectional study conducted on Japanese women [21,22], calorie and dietary fiber intake was not significantly different between subjects with FC and without FC. In addition, it has been reviewed that deficient fiber intake is not the main cause of constipation [11]. This is because there has not been a decrease in the prevalence of constipation in the United States over the last decades despite the increasing availability and ingestion of fiber-rich foods. Nevertheless, additional fiber intake improves constipation and its associated symptoms in some patients [23,24]. Thus the relationship between nutrients and FC need to be explored further.

**Table 3:** Nutrients intakes of the subjects

Variables	FC patients (n=52)	Control (n=52)
Total Calorie (kcal/day)	1876.2 ± 67.6^*^	1695.2 ± 51.9
Total protein (g/day)	74.3 ± 2.8	67.6 ± 2.3
Animal protein(g/day)	42.0 ± 2.3	39.9 ± 2.2
Plant protein (g/day)	32.3 ± 1.4^**^	27.8 ± 1.0
Total fat (g/day)	56.8 ± 2.2	52.4 ± 2.2
Animal fat(g/day)	30.0 ± 1.7	31.9 ± 1.8
Plant fat (g/day)	26.8 ± 1.4^***^	20.5 ± 1.0
Carbohydrate (g/day)	250.9 ± 10.0	226.1 ± 7.5
Dietary fiber (g/day)	15.9 ± 0.7	14.3 ± 0.5
Total calcium (mg/day)	434.7 ± 24.9	413.3 ± 22.4
Animal calcium (mg/day)	183.0 ± 15.4	198.0 ± 17.0
Plant calcium (mg/day)	243.2 ± 13.2^*^	199.5 ± 7.9
Phosphorous (mg/day)	955.8 ± 37.8	880.9 ± 31.9
Total iron(mg/day)	12.1 ± 0.7	10.9 ± 0.4
Animal iron (mg/day)	3.7 ± 0.2	3.7 ± 0.2
Plant iron (mg/day)	8.3 ± 0.7	7.2 ± 0.3
Sodium (mg/day)	3862.8 ± 188.1	3656.1 ± 141.1
Zinc (mg/day)	8.6 ± 0.4	8.1 ± 0.4
Vitamin A (㎍ RE/day)	663.4 ± 41.9	625.2 ± 28.6
Vitamin B_1_ (mg/day)	1.3 ± 0.1	1.3 ± 0.1
Vitamin B_2_ (mg/day)	1.2 ± 0.1	1.1 ± 0.1
Vitamin B_6_ (mg/day)	1.9 ± 0.1	1.8 ± 0.1
Niacin (mg NE/day)	16.9 ± 0.9	16.1 ± 0.8
Vitamin C (mg/day)	63.2 ± 4.3	60.6 ± 3.4
Folic acid (㎍/day)	190.6 ± 9.7	180.5 ± 7.1
Cholesterol (mg/day)	366.8 ± 23.0	338.1 ± 18.2

Values are mean ± SE; *; p<0.05, **:p<0.01, ***:p<0.001 (by Student’s t-test)

### Life stress scores

Average total life stress scores of FC patients and control group were 37.5 and 20.5, respectively (Table 4). Task-related stress scores were higher compared to interpersonal relationship stress scores in all of the subjects. There were significant differences between FC patients and control group not only in total life stress scores (p<0.01) but also in future problem (p<0.05), economy (p<0.05), problem (p<0.05) and value problem (p<0.05). These scores of FC patients were significantly higher compared to control group.

**Table 4:** Life stress scores of the subjects

Variables	FC patients (n=52)	Control (n=52)
Interpersonal relationship stress		
Faculty problem	4.3 ± 2.0	1.9 ± 0.7
Lover problem	4.2 ± 1.6	3.9 ± 1.6
Friend problem	0.6 ± 0.2	0.2 ± 0.1
Family problem	3.0 ± 1.0	1.1 ± 0.4
Task-related stress		
School grade problem	16.5 ± 3.0	10.1 ± 1.9
Future problem	16.7± 2.6^*^	9.4 ± 1.7
Economy problem	10.1 ± 2.7^*^	4.0 ± 1.2
Value problem	11.6 ± 2.4^*^	5.8 ± 1.4
Total score	37.5 ± 5.5^**^	20.5 ± 3.3

Values are mean ± SE; *; p<0.05, **:p<0.01, ***:p<0.001 (by Student’s t-test)

Although the definite cause of FC is unclear, psychological stress may affect FC through the brain-gut axis. Previous studies have reported an association between FC and psychological symptoms such as anxiety and depression in adults [25,26].

According to a survey of Korean college students [9], the stress scores of future problem, value problem and economy problem were higher compared to other stress categories. In this study, the above three noted problem scores of FC patients were significantly higher compared to control group. Therefore, it was required special concern about stress in college students in order to reduce constipation as well as psychological problems.

### Correlation between nutrients intakes including dietary taurine and life stress scores

Average total correlation coefficients between nutrients intake including dietary taurine and life stress scores are presented in Additional File 1. Significantly positive correlations were observed between school grade problems and intake of animal iron (p<0.05) and cholesterol (p<0.05), and between future problems and intake of total protein (p<0.05), phosphorous (p<0.01), animal iron (p<0.05) and cholesterol (p<0.05). There were significantly negative correlations between faculty problems and intake of animal iron (p<0.05), and between lover problems and intake of total iron (p<0.05), sodium (p<0.05), vitamin A (p<0.05), vitamin B_6_ (p<0.05) and vitamin C (p<0.05), and between family problems and intake of dietary fiber (p<0.05), plant calcium (p<0.05), vitamin B_6_ (p<0.05) and folic acid (p<0.05). There were no significant correlations between all stress scores and taurine. In the previous survey of Korean college students [9], there were no significant correlations between taurine and scores of life stress in male subjects which was consistent with our present results.

It has been reported that conflicting association exists between stress and food intake [27-29]. Chronic life stress seemed to be related with a greater preference for energy and nutrient-dense foods [30], and stress reduced food intake [31]. In this study, stress was positively or negatively related to various nutrients. Thus a further investigation is needed about the relationship between categories of stress and various nutrients.

## Conclusions

Life stress scores and intake of total calorie, plant protein, plant fat, carbohydrate and plant calcium of FC patients were significantly higher compared to control group. In addition, there were significantly positive correlations between future problems in life stress and intake of total protein, phosphorous, animal iron and cholesterol. However, there were significantly negative correlations between some categories of life stress such as faculty, lover and family problems and some micronutrients such as iron, sodium, vitamin A, vitamin B_6_, vitamin C and folic acid. Therefore, a further large-scale study or dietary supplement study is needed to obtain direct evidence about the correlation between life stress and nutrients intake including dietary taurine in FC patients.

## Abbreviations

FC: functional constipation; BMI: body mass index.

## Competing interests

The authors declare that they have no competing interests.

## Authors' contributions

JSY carried out the data collection, performed the statistical analysis and drafted the manuscript. JYP participated in data collection and analysis, KJC supervised the design and execution of the study. All authors read and approved the final manuscript.

## Supplementary Material

Additional file 1 DOC (Microsoft word) Click here for file
