# Supplementary material for: A case-control study on the dietary taurine intake, nutrient status and life stress of functional constipation patients in Korean male college students
Source: J Biomed Sci. 2010 Aug 24;17(Suppl 1):S41. doi: 10.1186/1423-0127-17-S1-S41 (PMC2994376; doi:10.1186/1423-0127-17-S1-S41)
Supplement: Additional file 1 — DOC (Microsoft word) [file 1423-0127-17-S1-S41-S1.doc]

| Variables | Faculty problem | Lover problem | Friend problem | Family problem | School grade problem | Future problem | Economy problem | Value problem | Total  score |
| --- | --- | --- | --- | --- | --- | --- | --- | --- | --- |
| Total Calorie (kcal/day) | -.110 | -.093 | -.051 | -.034 | .081 | .137 | -.008 | -.029 | .074 |
| Total protein (g/day) | -.120 | -.122 | -.025 | -.126 | .134 | .214^*^ | .-041 | .021 | .116 |
| Animal protein (g/day) | -.134 | -.114 | -.027 | -.094 | .162 | .200 | .-097 | .027 | .127 |
| Plant protein (g/day) | -.034 | -.074 | -.010 | -.120 | .014 | .130 | -.082 | -.002 | .036 |
| Total fat (g/day) | -.083 | -.059 | -.056 | -.139 | .027 | .077 | .010 | -.102 | -.014 |
| Animal fat (g/day) | -.130 | -.104 | -.006 | -.075 | .064 | .087 | .049 | -.051 | .013 |
| Plant fat (g/day) | .030 | .037 | -.088 | -.140 | -.039 | .016 | -.048 | -.107 | -.040 |
| Carbohydrate (g/day) | -.087 | -.127 | -.032 | -.124 | .042 | .143 | -.095 | -.018 | .041 |
| Dietary fiber (g/day) | -.141 | -.176 | -.030 | -.203^*^ | .022 | .125 | -.160 | -.036 | -.005 |
| Total calcium (mg/day) | .034 | -.044 | -.072 | -.106 | -.009 | .081 | -.094 | .036 | -.048 |
| Animal calcium (mg/day) | .116 | .026 | -.083 | -.009 | .043 | .114 | -.009 | .123 | .007 |
| Plant calcium (mg/day) | -.105 | -.127 | -.020 | -.226^*^ | -.081 | -.007 | -.175 | -.110 | -.107 |
| Phosphorous (mg/day) | -.081 | -.118 | -.009 | -.123 | .154 | .265^**^ | .010 | .079 | .131 |
| Total iron (mg/day) | -.133 | -.196^*^ | -.024 | -.164 | .023 | .117 | -.102 | -.088 | -.010 |
| Animal iron (mg/day) | -.198^*^ | -.097 | -.057 | -.011 | .199^*^ | .198^*^ | .030 | .000 | .132 |
| Plant iron (mg/day) | -.069 | -.180 | -.004 | -.179 | -.055 | .051 | -.126 | -.099 | -.064 |
| Sodium (mg/day) | -.166 | -.207^*^ | -.111 | -.153 | .067 | .128 | -.112 | -.067 | .006 |
| Zinc (mg/day) | -.133 | -.136 | -.002 | -.123 | .121 | .185 | -.039 | -.016 | .074 |
| Vitamin A (㎍RE/day) | -.128 | -.229^*^ | -.007 | -.144 | -.101 | .049 | -.186 | .015 | -.105 |
| Vitamin B_1_ (mg/day) | -.123 | -.135 | -.044 | -.181 | -.074 | -.025 | -.100 | -.091 | -.100 |
| Vitamin B_2_ (mg/day) | -.011 | -.080 | -.005 | -.082 | -.033 | .089 | -.033 | -.037 | -.012 |
| Vitamin B_6_ (mg/day) | -.143 | -.206^*^ | -.028 | -.206^*^ | .007 | .075 | -.119 | -.068 | -.044 |
| Niacin (mg NE/day) | -.150 | -.130 | -.016 | -.133 | .087 | .108 | .056 | -.050 | .054 |
| Vitamin C (mg/day) | -.079 | -.205^*^ | -.054 | -.184 | .148 | .092 | -.118 | -.081 | .017 |
| Folic acid (㎍/day) | -.112 | -.192 | .039 | -.223^*^ | .000 | .000 | -.182 | -.137 | -.097 |
| Cholesterol (mg/day | -.180 | -.183 | -.063 | -.104 | .203^*^ | .209^*^ | -.017 | -.051 | .105 |
| Taurine (mg/day) | -.0.1 | -.020 | -.090 | -.025 | .134 | .126 | -.072 | .121 | .120 |

Values are correlation coefficients. *; p<0.05, **:p<0.01, ***:p<0.001 (by Pearson’s correlation coefficient)
